# A World Allergy Organization international survey on physical activity as a treatment option for asthma and allergies

**DOI:** 10.1186/1939-4551-7-34

**Published:** 2014-11-27

**Authors:** Andre Moreira, Matteo Bonini, Ruby Pawankar, Sandra D Anderson, Kai-Håkon Carlsen, Christopher Randolph, William Silvers, William Storms, John M Weiler, Sergio Bonini

**Affiliations:** Hospital of São João and Faculty of Medicine, University of Porto, Porto, Portugal; Department of Public Health and Infectious Diseases, “Sapienza” University of Rome, Rome, Italy; Institute of Translational Pharmacology, National research Council, Rome, Italy; Div of Allergy, Department of Pediatrics, Nippon Medical School, Tokyo, Japan; Department of Respiratory and Sleep Medicine, Royal Prince Alfred Hospital, Sydney, Australia; Department of Paediatrics, Oslo University Hospital, Oslo, Norway; Center for Allergy, Asthma & Immunology, Waterbury, CT USA; National Jewish Hospital and Research Center, Denver, Colorado USA; The William Storms Allergy Clinic, Colorado, USA; CompleWare Corporation, North Liberty, Kragujevac, USA; Department of Internal Medicine, Second University of Naples, Naples, Italy

**Keywords:** Allergy, Asthma, Physical activity, Survey

## Abstract

**Background:**

Physical exercise has been shown to improve asthma symptoms, QoL, exercise capacity, bronchial hyperresponsiveness and lung function and is recommended as a supplementary treatment to pharmacotherapy for asthma. Clinicians are well placed to promote physically active lifestyles, but their role and practice towards promoting physically active lifestyles among patients has not been fully investigated. This study was designed to investigate the knowledge, propensity, attitude and practices of clinicians towards the promotion of physical activity among patients with asthma and allergies.

**Methods:**

Two hundred and eighty clinicians (mean age; 46 ± 13 years; with a clinical experience of practice for 15 ± 7 years) participated in a global survey. The survey comprised a 29-item questionnaire, which gathered information on attitudes of the clinicians towards promoting physical activity, their knowledge and their beliefs regarding evidence for benefits of physical activity as a supplementary treatment in patients with asthma and allergies.

**Results:**

Almost all respondents were aware of the strong evidence in favor of physical activity for the psychological well-being, weight control, decreased risk of diabetes, ischemic heart disease and arterial hypertension. Evidence for reduction in the risk for developing asthma and for better asthma control were reported by 60.0% and 85.4% of participants, respectively. The majority (85.0%) of clinicians strongly agreed that promoting physical activity is important to health care, although 95.5% considered they required more educational training. Although two thirds of them usually recommended exercise to their asthmatic/allergic patients, only 24.0% reported having previous training on the subject of such counseling. Almost all believed that effective counseling about a healthy diet, exercise and weight management would be easier if the clinician himself/herself was physically fit and healthy.

**Conclusions:**

The results of this global survey indicate that clinicians working in the field of allergy and respiratory diseases are well aware of the evidence supporting the benefits of physical activity for asthma and allergic diseases although they need more training in such counseling. Therefore, concerted efforts are needed towards educating clinicians towards promoting physical activity and weight management, as a supplementary treatment for asthma and allergies.

## Introduction

Changing dietary habits, sedentary lifestyle leading to reduced physical activity and obesity are distinct but strongly interrelated to lifestyle factors that may be relevant both to the development and management of asthma and allergic diseases. The integral role of physical activity and improved nutrition by healthier dietary patterns, such as Mediterranean diet e.g., has been shown to reduce the threat of chronic diseases [1]. Physical activity is a key determinant of energy expenditure, and thus fundamental to energy balance and weight control. However, the beneficial effects of physical activity are mediated by mechanisms beyond controlling excess body weight.

Non-pharmacological treatment approaches in asthma that focuses on interventions such as dietary habits and physical activity are of major interest. Such an approach could potentially reduce the dose requirements of pharmacological medications and reduce their side effects, improve quality of life and reduce the burden of disease. However, in real life, changing lifestyles by increased physical activity, preventing obesity and improving nutrition thereby enhancing health and well being is currently a public health challenge with benefits that extend beyond allergic diseases.

Therefore, this survey aims to assess the knowledge, attitude and practices of clinicians members of the World Allergy Organization (WAO) towards the promotion of physical activity among patients with asthma and allergic diseases.

## Methods

An original questionnaire was developed by and circulated among members of the WAO Special Committee on Sports and Allergy for preliminary evaluation. The questionnaire was designed to collect information on the respondent’s attitudes towards promoting physical activity, and on their knowledge, attitudes and beliefs about the existence of evidence on the beneficial effects of physical activity for various conditions. The final questionnaire comprised a total of 29 questions, and was approved by the committee and the WAO Board (Table 1).

**Table 1:** **Questionnaire distributed to participants**

***1. About you***					
1.1. Age, years					
1.2. Gender, female/male					
1.3. Country					
1.4. Your clinic practice is mainly in a…	Central Hospital	Outpatient clinic			
1.5. For how many years have you been an allergist?	Less than 5	5 to 10	More than 10		
1.6. In your practice do you manly see	Children	Adults	Both		
1.7. How many of them have asthma?					
2. ***Knowledge about existence of evidence of beneficial effects of physical activity***					
2.1. Improves the strength of bones and muscles	Strong evidence	Some evidence	No evidence	Do not know	
*2.2.* Improves psychological well-being	Strong evidence	Some evidence	No evidence	Do not know	
*2.3.* Helps in weight control	Strong evidence	Some evidence	No evidence	Do not know	
*2.4.* Reduces death from ischemic heart disease	Strong evidence	Some evidence	No evidence	Do not know	
*2.5.* Reduces the risk of hypertension	Strong evidence	Some evidence	No evidence	Do not know	
*2.6.* Reduces premature death	Strong evidence	Some evidence	No evidence	Do not know	
*2.7.* Reduces blood pressure in known hypertensive	Strong evidence	Some evidence	No evidence	Do not know	
*2.8.* Reduces the risk of non-insulin-dependent diabetes mellitus	Strong evidence	Some evidence	No evidence	Do not know	
*2.9.* Reduces the risk of breast cancer	Strong evidence	Some evidence	No evidence	Do not know	
*2.10.* Reduces asthma risk	Strong evidence	Some evidence	No evidence	Do not know	
*2.11.* Improves asthma control	Strong evidence	Some evidence	No evidence	Do not know	
*2.12.* Reduces allergic rhinitis incidence	Strong evidence	Some evidence	No evidence	Do not know	
*2.13.* Improves allergic rhinitis control	Strong evidence	Some evidence	No evidence	Do not know	
3. ***Attitudes toward promoting physical activity***					
3.1. Promoting physical activity is important in health care	Strongly agree	Agree	Neither agree nor disagree	Disagree	Strongly disagree
3.2. Advice to increase physical activity is more effective when linked to an individual’s presenting problem	Strongly agree	Agree	Neither agree nor disagree	Disagree	Strongly disagree
3.3. I can be effective in promoting health	Strongly agree	Agree	Neither agree nor disagree	Disagree	Strongly disagree
3.4. I can be effective in persuading some patients to increase physical activity	Strongly agree	Agree	Neither agree nor disagree	Disagree	Strongly disagree
3.5. I have sufficient knowledge to advise patients about physical activity	Strongly agree	Agree	Neither agree nor disagree	Disagree	Strongly disagree
3.6. Any amount of physical activity is beneficial to health	Strongly agree	Agree	Neither agree nor disagree	Disagree	Strongly disagree
3.7. Only vigorous/strenuous activity is beneficial to health	Strongly agree	Agree	Neither agree nor disagree	Disagree	Strongly disagree
3.8. I try to encourage as many patients as possible to increase their physical activity	Strongly agree	Agree	Neither agree nor disagree	Disagree	Strongly disagree
3.9. I only discuss physical activity if the patient mentions it	Strongly agree	Agree	Neither agree nor disagree	Disagree	Strongly disagree
4. ***Attitudes and beliefs toward promoting physical activity as part of disease management***					
4.1. With a typical asthmatic patient, how often do you actually talk about exercise?	Never or rarely	Sometimes	Usually or always		
4.2. How relevant do you think talking to asthmatic patients about exercise will be in your intended practice?	Not at all	Somewhat	Highly		
4.3. How much training have you had on talking to asthmatic patients about exercise?	None	Some	Extensive		
4.4. Doctors need more training in preventive care	Strongly agree	Agree	Neither agree nor disagree	Disagree	Strongly disagree
4.5. I will be able to provide more credible and effective counseling if I eat a healthy diet	Strongly agree	Agree	Neither agree nor disagree	Disagree	Strongly disagree
4.6. I will be able to provide more credible and effective counseling if I exercise and stay fit	Strongly agree	Agree	Neither agree nor disagree	Disagree	Strongly disagree
4.7. I will be able to provide more credible and effective counseling if I maintain a healthy weight	Strongly agree	Agree	Neither agree nor disagree	Disagree	Strongly disagree

It is our hope that the results of this survey facilitates collaboration and education among the different allergy centres. Please check all answers that apply.

The objective of this is to survey the knowledge, attitudes and self-reported practice of allergists towards promoting regular physical activity as part of non-pharmacological treatment to asthma and allergic conditions.

The questionnaire was then converted into a web-based format and distributed electronically to clinician members of the 92 member and regional societies of WAO. Representatives of member societies were asked to respond or delegate to the most appropriate expert who could appropriately answer the specific questions. All respondents were given a period of eight weeks to reply. Technical experts in the WAO Secretariat then collated the responses. Descriptive statistics of frequency, percentages was used to summarize the data.

## Results

**Figure 1:**
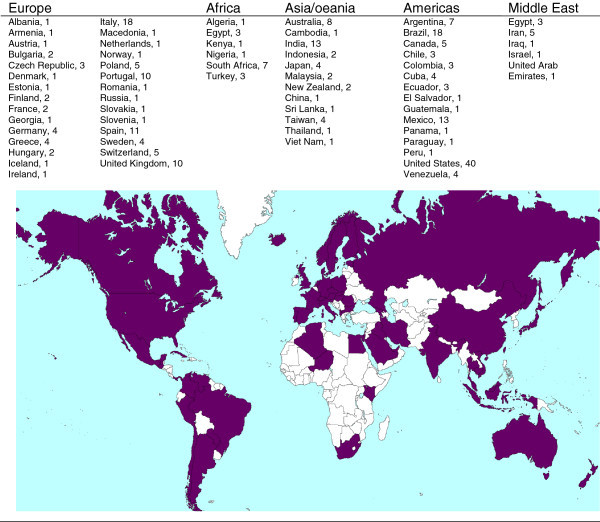
**Geographical distribution of clinicians participating in the survey.**

There was a total of 280 responses from WAO member societies. The geographical distribution of all respondents is presented in Figure 1. The average age of the respondents was 46 ± 13 years, and their average length of being in clinical practice was 15 ± 7 years. Demographic characteristics and profiles of respondent clinicians, by medical specialty, are as shown in Table 2. Allergy and/or clinical immunology were the most represented medical disciplines among responders (n = 209, 74.6%).

**Table 2:** **Characteristics of responders by** ***medical specialty***

	Allergy and clinical immunology	Dermatology	ENT	Pediatrics	Respiratory	General medicine*	Total
	n = 209	n = 4	n = 6	n = 26	n = 23	n = 12	n = 280
Your clinic setting is mainly							
Outpatient	95 (45.5)	1 (25.0)	1 (16.7)	12 (46.2)	4 (17.4)	8 (66.7)	121 (43.2)
University	80 (38.3)	1 (25.0)	4 (66.7)	9 (34.6)	13 (56.5)	3 (25.0)	110 (39.3)
Private	34 (16.3)	2 (50.0)	1 (16.7)	5 (19.2)	6 (26.1)	1 (8.3)	49 (17.5)
For how many years have you been an allergist?	15 ± 7	21 ± 0	16 ± 9	14 ± 8	18 ± 12	9 ± 6	15 ± 7
Does your practice mainly see**							
Children	33 (15.9)	0	0	23 (88.5)	2 (9.1)	1 (8.3)	59 (21.3)
Adults	37 (17.8)	0	2 (33.3)	0	14 (63.6)	3 (25.0)	56 (20.2)
Both	138 (66.3)	3 (100)	4 (66.7)	3 (11.5)	6 (27.3)	8 (66.7)	162 (58.5)
How many have asthma? mean ± sd	30 ± 9	25 ± 0	25 ± 0	29 ± 9	33 ± 12	28 ± 7	30 ± 9

Data presented n (%) unless otherwise stated; ENT: ear, nose and throat; *includes Internal Medicine and Sports Medicine; **p < .0001, chi-square.

Concerning knowledge on the beneficial effects of physical activity, the majority was aware of a strong evidence supporting improvements in the strength of bones and muscles, psychological well-being, weight control, as well as lowering the risk of non-insulin-dependent diabetes mellitus, death from ischemic heart disease and hypertension (Table 3). Almost half of the responders, 47.9% and 54.3% respectively, reported some evidence for reduction in the risk of developing asthma and for improvement of asthma control. The majorities believe there is no evidence for physical activity to reduce the incidence or to improve allergic rhinitis (Table 3).

**Table 3:** **Knowledge about evidence of beneficial effects of physical activity for various conditions**

Statement regarding condition	Strong evidence	Some evidence	No evidence	Did not know
	n (%)	n (%)	n (%)	n (%)
Improves the strength of bones and muscles	225 (80.4)	49 (17.5)	2 (0.7)	4 (1.4)
Improves psychological well-being	212 (75.7)	64 (22.9)	3 (1.1)	1 (0.4)
Helps in weight control	224 (80.0)	52 (18.6)	2 (0.7)	2 (0.7)
Reduces death from ischemic heart disease	156 (55.7)	106 (37.9)	9 (3.2)	9 (3.2)
Reduces the risk of hypertension	156 (55.7)	108 (38.6)	9 (3.2.)	7 (2.5)
Reduces premature death	96 (34.3)	129 (46.1)	23 (8.2)	32 (11.4)
Reduces blood pressure in known hypertensive	133 (47.5)	113 (47.5)	17 (6.1)	15 (5.4)
Reduces the risk of non-insulin-dependent diabetes mellitus	153 (54.6)	96 (34.3)	16 (5.7)	14 (5.0)
Reduces the risk of breast cancer	31 (11.1)	91 (32.5)	78 (27.9)	80 (28.6)
Reduces asthma risk	34 (12.1)	134 (47.9)	70 (25.0)	42 (15)
Improves asthma control	87 (31.1)	152 (54.3)	22 (7.9)	19 (6.8)
Reduces allergic rhinitis incidence	17 (6.1)	68 (24.3)	129 (46.1)	66 (23.6)
Improves allergic rhinitis control	28 (10)	85 (30.4)	108 (38.6)	59 (21.1)

Eighty five percent of responders strongly agreed that promoting physical activity is important in general in health care, and 48.9% responded that advice to increase physical activity is more effective when linked to an individual’s disease itself (Table 4). Almost half (45.7%) considered themselves as having sufficient knowledge to advise patients about physical activity and reported that they try (45.4%) to encourage as many patients as possible to increase their physical activity.

**Table 4:** **Attitudes towards promoting physical activity**

Statement regarding condition	Strongly agree,	Agree	Neither agree nor disagree	Disagree	Strongly disagree
	n (%)	n (%)	n (%)	n (%)	n (%)
Promoting physical activity is important in health care	238 (85)	39 (13.9)			
Advice to increase physical activity is more effective when linked to an individual’s presenting problem	137 (48.9)	119 (42.5)	14 (5.0)	5 (1.8)	
I can be effective in promoting health	137 (48.9)	126 (45)	13 (4.6)		
I can be effective in persuading some patients to increase physical activity	116 (41.4)	136 (48.6)	23 (8.2)		
I have sufficient knowledge to advise patients about physical activity	82 (29.3)	128 (45.7)	51 (18.2)	10 (3.6)	2 (0.7)
Any amount of physical activity is beneficial to health	115 (41.1.)	112 (40)	21 (7.5)	23 (8.2)	3 (1.1)
Only vigorous/strenuous activity is beneficial to health	10 (3.6)	20 (7.1)	51 (18.2)	151 (53.9)	43 (15.4)
I try to encourage as many patients as possible to increase their physical activity	116 (41.4)	127 (45.4)	24 (8.6)	8 (2.9)	1 (0.4)
I only discuss physical activity if the patient mentions it	13 (4.6)	38 (13.6)	36 (12.9)	132 (47.1)	54 (19.3)

Two thirds of the responders either usually or always talked about exercise with their asthmatic patients, although only 24% reported that they had had previous training on such counseling. Almost all (95.5%) responded that doctors need more training in counseling with regards to preventative care. Moreover, the majority believed that effective counseling regarding a healthy diet, exercise and weight management would be easier if doctors themselves were physically fit and healthy (Table 5a and 5b).

**Table 5:** **Attitudes and beliefs towards promoting physical activity as part of disease management**

Statement regarding condition					
With a typical asthmatic patient, how often do you actually talk about exercise?		Never or rarely: 12 (4.4)	Sometimes: 85 (31.1)	Usually or always: 176 (64.5)	
How relevant do you think talking to asthmatic patients about exercise will be in your intended practice?		Not at all: 8 (3.0)	Somewhat: 95 (35.3)	Highly: 166 (61.7)	
How much training have you had on talking to asthmatic patients about exercise?		None: 90 (33.2)	Some: 116 (42.8)	Extensive: 65 (24.0)	
Data presented as n (%)					
**Statement regarding condition**	**Strongly agree**	**Agree**	**Neither agree nor disagree**	**Disagree**	**Strongly disagree**
	**n (%)**	**n (%)**	**n (%)**	**n (%)**	**n (%)**
Doctors need more training in preventive care	151 (55.1)	111 (39.6)	9 (3.2)	2 (0.7)	1 (0.4)
I will be able to provide more credible and effective counseling if I eat a healthy diet	103 (36.8)	128 (45.7)	29 (10.4)	13 (4.6)	3 (1.1)
I will be able to provide more credible and effective counseling if I exercise and stay fit	116 (42.2)	129 (46.9)	21 (7.6)	8 (2.9)	1 (0.4)
I will be able to provide more credible and effective counseling if I maintain a healthy weight	117 (42.4)	135 (48.9)	19 (6.9)	5 (1.8)	0 (0)

## Discussion

This study reports the knowledge, attitude and practice of clinicians towards the promotion of physical activity to achieve better health in the management of patients with asthma and allergies in conjunction with pharmacotherapy. Our survey demonstrates that the majority of allergists are aware of the existence of the strong evidence in favour of physical activity for psychological well being, weight control, and decreased risk of diabetes, ischemic heart disease and hypertension. Evidence for reduction of the risk for the development of asthma and for better asthma control was reported by 60% and 85.4% respectively. The majority strongly agreed that promoting physical activity is important to health care, although almost all recommended the need for more training for doctors in preventative care. Finally, almost all of the respondents agreed that effective counseling regarding a healthy diet; exercise and weight management would be easier if oneself remained fit and healthy.

The study has some limitations. First, it is not possible to exclude a selection bias. About forty percent of the responders worked in a University setting and may be particularly aware of the importance of physical activity in disease management. It was also not possible to assess response rate, as we are not aware of how many recipients got the survey email. We could therefore infer that the results may not be fully representative of current practice. Yet, even in these potentially selected participants, there seems to be room to improve promotion of physical activity to their patients as well as emphasizing it in the curriculum. Secondly, the questionnaire used in collecting data was neither pre-tested, nor validated. Nonetheless, the contents in the questionnaire were well understood by the respondents.

The evidence supporting the health benefits at the population level of diet and exercise throughout the course of life is indisputable [1]. Allergies envolve systemic disease affecting multiple organs and the severity and complexity of allergies are increasing, especially in children [2]. Clinicians are in a unique position for tailoring information and they can play a major role in motivating patients towards a healthy life style change that could have benefits to other non-communicable diseases also beyond allergies. Unhealthy weight gain can be prevented by increasing physical activity, and by reducing intakes of foods that are rich in fat, and foods and drinks rich in sugar content. There is evidence that obesity both increases the risk of incidence of asthma and worsens the severity of asthma towards a more difficult-to-control disease. In a recent study, looking at the studies reporting an effect of weight change on people who have asthma, it was shown that becoming obese doubled the risk of being asthmatic [3]. The authors however found little evidence that weight reduction had a positive impact on asthma. Nevertheless, as benefits extend much further than asthma, targeting weight in overweight or obese asthmatics should be recommended as part of their management [4, 5].

In 2010, about one out of three adults (32.4%) of a representative sample of the civilian non-institutionalized population of the United States who had seen a physician in the past year had been advised to exercise or do other physical activity [6]. Furthermore, adults who were obese were about twice as likely as healthy weight adults to have been advised. Also, the percentage of adults who had been advised by their physician to exercise increased with age from the youngest [6]. Across the chronic health conditions studied, adults with diabetes were the most likely, and those with cancer were the least likely, to have been advised by their physician to exercise. This is fairly in agreement with our observations where half of the responders considered they had sufficient knowledge to advise patients about physical activity and tried to encourage as many patients as possible to increase their physical activity. Still, even in our possible biased responders the proportion of patients receiving this advice remains well below the desirable level.

Studies have demonstrated a positive association between personal physical activity habits of physicians and their counseling behavior’s [7]. In the present survey, about two thirds of the allergists considered their counseling would be more credible and effective if they themselves were fit, and had a healthy weight and diet. This observation is particularly important as physicians are regarded as faithful, highly credible members of the populations they care for. This delivers not only an opportunity, but also a duty to promote a physically and a healthy active lifestyle.

As in previous studies, [8, 9] clinicians reported that they need more education on the benefits of physical activity, although they consider themselves effective in promoting health and persuading patients to increase their physical activity. Knowledge about how to use exercise as a supplementary treatment and how to develop strategies that promote behavioural change is ‘sparse or non-existent’ in the medical curriculum in many countries. For this reason changes should be made to provide allergists with the knowledge and skills necessary to promote physical activity effectively.

In conclusion, the information provided by this first worldwide survey on allergists practice towards physical activity supports the need for education and training regarding physical activity and weight management. This training should be part of the treatment modalities for allergic diseases across WAO member societies. Therefore, we propose the following action plan: (1) to develop and include educational training for allergists to effectively counsel about physical activity to their patients; (2) to encourage allergists to act as physical active role models in their communities; and finally, (3) to promote clinical research and to interest policy-makers and public health professionals to develop and support larger and better-designed intervention studies to look at the effect of physical activity in asthma.

## References

[1] WHO (2003). Diet, nutrition and the prevention of chronic diseases. World Health Organ Tech Rep Ser.

[2] Pawankar R (2012). The unmet global health need of severe and complex allergies: meeting the challenge. World Allergy Organ J.

[3] Moreira A, Bonini M, Garcia-Larsen V, Bonini S, Del Giacco SR, Agache I, Fonseca J, Papadopoulos NG, Carlsen KH, Delgado L, Haahtela T (2013). Weight loss interventions in asthma: EAACI evidence-based clinical practice guideline (part I). Allergy.

[4] Carson KV, Chandratilleke MG, Picot J, Brinn MP, Esterman AJ, Smith BJ: **Physical training for asthma.** *Cochrane Database Syst Rev* 2013, [Internet] 2013 [cited 2014 Mar 12]; 9: CD001116Available from: http://www.ncbi.nlm.nih.gov/pubmed/24085631 10.1002/14651858.CD001116.pub4 PMC11930393 24085631

[5] Eichenberger PA, Diener SN, Kofmehl R, Spengler CM (2013). Effects of exercise training on airway hyperreactivity in asthma: a systematic review and meta-analysis. Sports Med.

[6] Barnes PM, Schoenborn CA (2012). Trends in adults receiving a recommendation for exercise or other physical activity from a physician or other health professional. NCHS Data Brief.

[7] Services USDOHAH: **Physical Activity and Health: A Report of the Surgen General.** *Rev Prat* 2010,. 996 Available from: http://www.ncbi.nlm.nih.gov/pubmed/20467523

[8] McKenna J, Naylor PJ, McDowell N (1998). Barriers to physical activity promotion by general practitioners and practice nurses. Br J Sports Med.

[9] Tulloch H, Fortier M, Hogg W (2006). Physical activity counseling in primary care: who has and who should be counseling?. Patient Educ Couns.

